# *Anaplasma phagocytophilum* Infection in Small Mammal Hosts of *Ixodes* Ticks, Western United States

**DOI:** 10.3201/eid1407.071599

**Published:** 2008-07

**Authors:** Janet E. Foley, Nathan C. Nieto, Jennifer Adjemian, Haydee Dabritz, Richard N. Brown

**Affiliations:** * University of California, Davis, California, USA; † California Department of Public Health, Richmond, California, USA; ‡ Humboldt State University, Arcata, California, USA

**Keywords:** anaplasmosis, disease ecology, reservoir, dispatch

## Abstract

A total of 2,121 small mammals in California were assessed for *Anaplasma phagocytophilum f*rom 2006 through 2008. Odds ratios were >1 for 4 sciurids species and dusky-footed woodrats. High seroprevalence was observed in northern sites. Ten tick species were identified. Heavily infested rodent species included meadow voles, woodrats, deer mice, and redwood chipmunks.

*Anaplasma phagocytophilum* is a tick-transmitted pathogen that causes granulocytic anaplasmosis in humans, horses, and dogs (*1*–*3*). *A. phagocytophilum* is maintained in rodent-*Ixodes* spp. tick cycles, including the western black-legged tick (*Indopacetus* *pacificus*) in the western United States (*4*). Transovarial transmission does not occur, and *I.* *pacificus* feeds only 1 time per stage, so infection must be acquired by a juvenile tick feeding on an infected mammal. Suggested reservoirs in the West include the dusky-footed woodrat (*Neotoma fuscipes)*, for which chronic infection has been observed, and the western gray squirrel (*Sciurus griseus*), which are frequently infected in nature (*5*,*6*). The northern coast range and Sierra Nevada foothills of California (*4*,*7*), where abundant rodents include deer mice (*Peromyscus* spp.), woodrats, and chipmunks (*Tamias* spp.), have moderate to high levels of granulocytic anaplasmosis. We sought to evaluate granulocytic anaplasmosis exposure and infection and describe the *Ixodes* spp. tick fauna in small mammals from central and northern coastal California.

## The Study

Small mammals were caught in live traps (HB Sherman, Tallahassee, FL, USA, and Tomahawk Live Trap, Tomahawk, WI, USA) at 9 sites or collected as carcasses on roads (Technical Appendix) from 2006 to 2008. Traps were set at locations of observed active rodent use or dens and baited with peanut butter and oats or corn, oats, and barley. Rodents were anesthetized with ketamine and xylazine delivered subcutaneously, examined for ectoparasites, and bled by retro-orbital abrasion or femoral venipuncture. The blood was anticoagulated with EDTA. Shrew (*Sorex* spp.) carcasses were retrieved when found in traps, kept cold, and then sampled in the laboratory. Live shrews were examined for ticks but released without further processing. All carcasses were identified to species, age, and sex; examined for ectoparasites; and then dissected for coagulated heart blood and spleen. Ectoparasites were preserved in 70% ethanol for identification. Data were included for animals from 3 previous studies (*5*,*8*,*9*).

Plasma anti–*A. phagocytophilum* immunoglobulin (Ig) G was assayed by an indirect immunofluorescent antibody assay (*3*), by using *A. phagocytophilum*–infected HL-60 cells as substrate and fluorescein isothiocyanate–labeled goat anti-rat heavy and light chain IgG (Kirkegaard and Perry, Gaithersburg, MD, USA). This assay does not distinguish exposure to *A. phagocytophilum* from *A. platys,* but the PCR was specific for *A. phagocytophilum.* PCR was performed for all flying (*Glaucomys sabrinus*), Douglas (*Tamiasciuris douglasii)*, and gray squirrels; all chipmunks from Santa Cruz and Marin Counties; a random subset of chipmunks from Humboldt Redwoods State Park and Hendy Woods State Park; and a random subset of individual mammals of other species. DNA was extracted from whole blood by using a kit (DNeasy Tissue kit, QIAGEN, Valencia, CA, USA), and real-time PCR was performed as described previously (*5*).

Data were analyzed with “R” (www.r-project.org), with a cutoff for statistical significance of p = 0.05. Differences in seroprevalence among small mammal species and between sexes were assessed by χ^2^ test. Individual small mammals’ risk for *A. phagocytophilum* exposure and infection were assessed as a function of sex, species, and location by calculating odds ratios (OR) and 95% confidence intervals (CI). Multivariate logistic regression was performed to evaluate seropositivity as a function of site, host species, and interactions to evaluate possible interaction and confounding between the variables.

A total of 2,121 small mammals, including 2,100 rodents, 20 shrews, and 1 lagomorph, were evaluated for exposure to and infection with *A. phagocytophilum* and infestation with *Ixodes* spp. ticks (Table 1). The overall seroprevalence was 15.2% (95% CI 13.6–16.9). Highest values and ORs >1 occurred in dusky-footed woodrats, tree squirrels, and some chipmunk species (Table 1; Technical Appendix). The PCR prevalence among rodents tested was 3.8% (N = 652, 95% CI 2.9–5.3); highest values were reported in tree squirrels and some chipmunk species (Table 1). Although deer mice have been reported to be exposed to *A. phagocytophilum* (*10*,*11*), we found little evidence of this in our study. Woodrats at northern sites tended to be infected, while sciurids (excluding ground squirrels) showed high rates of exposure at multiple sites, consistent with previous reports (*5*). A total of 60% of eastern gray squirrels from Connecticut were seropositive with reservoir competence documented by producing PCR-positive ticks after feeding on infected squirrels (*12*). A PCR-positive eastern chipmunk (*Tamias striatus*) was reported from Minnesota (*13*).

**Table 1:** Seroprevalence and PCR prevalence of *Anaplasma phagocytophilum* among small mammal species, northern and central coastal California*

Mammal species	*A. phagocytophilum* IFA				*A. phagocytophilum* msp2 PCR		
	Seropositive	Seroprevalence	95% CI		PCR positive	PCR prevalence	95% CI
*Clethrionomys californicus*	1	12.50	0.6–53.3		0	0	0–53.7
*Glaucomys sabrinus*	2	14.29	2.5–43.9		1	16.7	0.8–63.5
*Mus musculus*	0	0.00	0–25.3		0	0	0–34.4
*Microtus californicus*	2	5.88	1.0–21.1		0	0	0–17.8
*Neotoma cinerea*	0	0.00	0–94.5		0	0	0–94.5
*N. fuscipes*	167	50.15	44.7–55.6		8	4.3	2.0–8.6
*N. macrotis*	2	3.03	5.3–11.5		1	1.8	0.09–10.6
All *Neotoma*	169	42.25	37.4–47.3		9	3.7	1.8–7.1
*Peromyscus boylii*	3	8.82	2.3–24.8		1	4.0	0.2–22.3
*P. californicus*	2	0.67	0.1–2.7		0	0	0–3.8
*P. maniculatus*	18	3.46	2.1–5.5		0	0	0–6.6
*P. truei*	1	2.56	0.1–15.1		NT		
*Peromyscus* spp.	0	0.00	0–53.7		NT		
All *Peromyscus*	24	2.68	1.8–4.0		1	0.45	0.02–2.9
*Rattus rattus*	0	0.00	0–37		0	0	0–37.1
*Reithrodontomys megalotis*	0	0.00	0–17.2		1	6.3	0.3–32.3
*Spermophilus beecheyi*	0	0.00	0–4.2		0	0	0–20.0
*S. lateralis*	2	22.22	3.9–59.9		NT		
*Sciurus carolinensis*	11	57.89	34.0–78.9		3	18.8	5.0–46.3
*S. griseus*	34	70.83	55.7–82.6		6	15.8	6.6–31.9
*S. niger*	1	100.00	55.0–100.0		0	0	0–94.5
All *Sciurus*	46	47.83	33.1–62.9		9	16.4	8.2–29.3
*Sorex* spp.	0	0.00	0–37.0		0	0	0–94.5
*Sylvilagus bachmani*	0	0.00	0–94.5		NT		
*Tamias amoenus*	6	6.82	2.8–14.8		NT		
*T. merriami*	0	0.00	0–48.3		0	0	0–40.2
*T. minimus*	0	0.00	0–4.9		NT		
*T. senex*	5	4.81	1.8–11.4		NT		
*T. speciosus*	4	33.33	11.3–64.6		NT		
*T. sonomae*	1	14.29	0.7–58.0		2	50.0	15.0–85.0
*T. ochrogenys*	30	27.52	19.6–37.0		2	6.9	1.2–24.2
*Tamias* spp.	2	8.33	1.5–28.5		NT		
All *Tamias*	48	13.45	10.2–17.5		4	34.0	3.2–24.1
*Tamiasciurus douglasii*	6	40.00	17.5–67.1		0	0	0–60.4
Total	300	15.24	13.7–16.9		33	3.8	2.9–5.3

*IFA, immunofluorescence assay; CI, confidence interval; NT, not tested.

Location was an important determinant of exposure to infection, with high seroprevalence in the Hoopa Valley Indian Reservation and Hendy Woods State Park (Table 2). ORs significantly <1 were observed for Samuel P. Taylor State Park and the Morro Bay area, and 5 sites in the far northern coast range and Quincy in the Sierra Nevada had ORs >1 (Technical Appendix). Statistical analysis failed to document a significant interaction between site and host species, but confounding was apparent, with overrepresentation of gray squirrels and woodrats in some high prevalence sites (Technical Appendix). PCR prevalence was high at Sutter Buttes State Park and Siskiyou County (both with low sample size) and Big Basin State Park and Hendy Woods State Park, each ≈12% (Table 2). Results are consistent with prior reports for horses and dogs (*4*). Previous spatial analysis documented increased *A. phagocytophilum* risk in redwood, montane hardwood, and blue oak/foothill pine habitats (*14*). In our dataset, obvious habitat differences would not account for differences in disease exposure, given the presence of live oak, tanoak, redwood, and Douglas fir at many sites. Further ecologic studies to identify differing ecologic factors among these sites would be useful.

**Table 2:** Regional seroprevelance and PCR prevalence rates for exposure to *Anaplasma phagocytophilum* in small mammals in various sites, northern and central California*

Site	*A. phagocytophilum* IFA				*A. phagocytophilum* msp2 PCR		
	Seropositive	Seroprevalence	95% CI		PCR positive	PCR prevalence	95% CI
Big Basin State Park	16	6.30	3.76–10.22		5	12.20	4.58–27.00
Humboldt Redwoods State Park	24	16.90	11.33–24.31		2	6.06	1.06–21.62
Hoopa Valley Indian Reservation	173	36.19	31.91–40.70		6	4.14	1.69–9.18
Hendy Woods State Park	43	22.51	16.93–29.22		5	12.19	4.58–27.00
King Range National Conservation Area	1	3.45	0.18–19.63		0	0.00	0.00–80.21
Mendocino County (roadside only)	0	0.00	0.00–94.53		0	0.00	0.00–94.54
Morro Bay regional communities	5	1.23	0.45–3.01		2	0.67	0.12–2.65
Placerville City region (roadside only)	1	1.00	5.46–1.00		1	1.00	5.46–1.00
Quincy City region (roadside only)	2	50.00	15.00–84.99		0	0.00	0.00–60.42
Sutter Buttes State Park	3	7.50	1.96–21.48		1	50.00	9.45–90.55
Sagehen Research Station	17	7.69	4.68–12.24		0	0.00	0.00–60.42
Siskiyou County (roadside only)	3	1.00	30.99–1.00		1	33.33	1.76–87.47
Sonoma	1	1.00	5.46–1.00		0	0.00	0.00–94.54
Samuel P. Taylor State Park	3	1.75	0.42–5.45		2	4.26	0.74–15.73
Trinity County (roadside only)	2	40.00	7.26–82.96		0	0.00	0.00–53.71
Sacramento River Valley (roadside only)	3	1.00	30.99–1.00		0	0.00	0.00–69.00
Willow Creek Town (roadside only)	3	0.30	8.09–64.63		0	0.00	0.00–60.42
Yolo County	1	6.67	0.35–33.97		0	0.00	0.00–25.35

*IFA, immunofluorescence assay; CI, confidence interval.

Tick species observed in our study sites include possible enzootic vectors and several human-biting species, including *I. pacificus* and *I. angustus* (Technical Appendix). Host species from which relatively large collections were obtained included meadow voles, woodrats, deer mice, tree squirrels, and redwood chipmunks (*T. ochrogenys)*. Tick diversity was highest on redwood chipmunks and in more northerly sites (Technical Appendix). *I. angustus,* primarily a nidicolous tick of rodents but occasionally bites humans and is a competent vector for *Borrelia burgdorferi* sensu stricto (*15*), occurred on most rodent species. *I. spinipalpis,* which occurred on woodrats, deer mice, squirrels, and chipmunks, functions as a primary vector for *B. bissettii* in a woodrat enzootic cycle (*16*), and *Neotoma mexicana* and *I. spinipalpis* have an enzootic cycle in Colorado for *A. phagocytophilum*.

## Conclusions

We show that a strong distinction can be made in possible reservoir capacity among rodent species, with many, such as deer mice and voles, only contributing to the ecology of granulocytic anaplasmosis through their support of ticks but not *A. phagocytophilum* infection. Others, including tree squirrels and woodrats, are frequently infected, in addition to supporting ticks. Considerable similarities exist between the ecology of *A. phagocytophilum* and *B. burgdorferi* in the West, although the large diversity of genospecies that exists for *B. burgdorferi* has not been reported for *A. phagocytophilum.* These data provide a starting point for future work to clarify the reservoir competence of small mammals for *A. phagocytophilum* and to determine how ecologic interactions among small mammals, other vertebrate hosts, multiple possible vectors, and both *B. burgdorferi* and *A. phagocytophilum* could affect the enzootic persistence of these pathogens and risk to humans and animals.

## Supplementary Material

Technical Appendix Anaplasma phagocytophilum Infection in Small Mammal Hosts of Ixodes Ticks, Western United States
