# Supplementary material for: Anaplasma phagocytophilum Infection in Small Mammal Hosts of Ixodes Ticks, Western United States
Source: Emerg Infect Dis. 2008 Jul;14(7):1147–50. doi: 10.3201/eid1407.071599 (PMC2600359; doi:10.3201/eid1407.071599)
Supplement: Technical Appendix — Anaplasma phagocytophilum Infection in Small Mammal Hosts of Ixodes Ticks, Western United States [file 07-1599_Techapp-s1.pdf]

Anaplasma phagocytophilum Infection in Small Mammal Hosts of *Ixodes* Ticks, Western United States

Technical Appendix

Table 1. Study sites evaluated for granulocytic anaplasmosis in wild rodents, northern and central coastal California

Study site	Dominant vegetation	Region	Latitude and longitude (decimal degrees)	Elevation (m)	Distance to coast (km)
Big Basin State Park	Redwood, live oak, tanoak, madrone, chaparral (1 site only)	Central coast range	37.10.621; 122.12.328	368	21
Humboldt Redwoods State Park	Redwood, Douglas fir, tanoak, madrone, live oak	Northern coast range	40.17.770; 123.59.178	610	26
Hoopa Valley Indian Reservation	Douglas fir, tanoak, madrone	Northern coast range	41.10.333; 123.56.520	109–1200	26
King Range National Conservation Area	Douglas fir, tanoak, madrone, redwood (pockets), live oak	Northern coast range	40.08.059; 124.07.404	61–610	3.9
Mendocino	peri-urban, live oak, Douglas fir	Northern coast range	39.40.050; 123.30.296	555	41
Morro Bay	Live oak, Monterey pine, eucalyptus	Central coast range	35.22.088; 120.49.464	61	1.6
Placerville	Douglas fir, Jeffrey pine, incense cedar	Sierra Nevada foothills	38.45.899; 120.49.464	1189	206
Quincy	Jeffrey pine, Douglas fir, incense cedar	Sierra Nevada	39.53.408; 120.51.312	1400	265
Sutter Buttes State Park	Blue oak, live oak,	Central valley butte	39.12.805; 121.48.167	247	151
Sagehen Research Station	Jeffrey pine, pine, red fir, white fir	Sierra Nevada	39.44.000; 120.22.000	1950	315
Siskiyou	Douglas fir, tanoak, madrone	Klamath	41.18.077; 123.32.296	198	48
Sonoma	Live oak	Northern coast range	38.19.169; 122.36.711	274	34
Samuel P. Taylor State Park	Redwood, live oak, tanoak, madrone	Northern coast range	38.01.232; 122.40.774	134	11
Trinity	Douglas fir, tanoak, madrone	Klamath	40.44.286; 123.03.222	488	131
Sacramento Valley	peri-urban	Central valley	38.33.481; 121.25.694	9	130
Willow Creek	peri-urban, Douglas fir, tanoak, madrone	Northern coast range	40.56.302; 123.37.615	229	41
Yolo	Live oak, blue oak, foothill pine	Northern coast range	38.49.382; 122.12.250	137	121

Table 2. Risk factors for *Anaplasma phagocytophilum* seropositivity with statistically significant odds ratios*

Risk factor	Odds ratio	95% CI lower	95% CI upper	p value
Sciurus carolinensis	14.01	3.15	62.40	5.0×10^{-4}
Sciurus griseus	23.43	6.19	88.66	3.5×10^{-6}
Neotoma fuscipes	10.38	3.11	34.65	1.4×10^{-4}
Peromyscus californicus	0.07	0.01	0.43	4.2×10^{-3}
Tamiasciuris douglasii	6.89	1.43	33.18	0.016
Tamias ochrogenys	3.92	1.11	13.81	0.033
Humboldt Redwoods	3.16	1.55	6.42	2.6×10^{-3}
Hoopa Valley	3.25	1.77	5.97	1.5×10^{-4}
Hendy Woods	4.37	2.28	8.36	8.3×10^{-6}
Morro Bay	0.19	0.07	0.54	1.7×10^{-3}
Quincy	29.64	2.53	347.50	6.9×10^{-3}
Samuel P. Taylor	0.28	0.08	0.98	0.047
Trinity	15.17	1.98	115.99	8.8×10^{-3}
Willow Creek	7.06	1.58	31.52	0.01

*CI, confidence interval.

Table 3. Proportion of all hosts collected from sites in northern and central California represented by *Sciurus* spp. and *Neotoma* spp.

Location	Animals tested	Tree squirrels tested	Proportion of animals that were squirrels	Woodrats tested	Proportion of animals that were woodrats
Big Basin State Park	254	21	8.3	25	9.8
Humboldt Redwoods State Park	142	5	3.5	8	5.6
Hoopa Valley Indian Reservation	478	12	2.5	273	56.1
Hendy Woods State Park	191	8	4.2	20	10.5
King Range National Conservation Area	29	1	3.4	0	0
Mendocino County (roadside only)	1	1	100.0	0	0
Morro Bay regional communities	407	0	0	40	9.8
Placerville City region (roadside only)	1	1	100.0	0	0
Quincy City region (roadside only)	6	3	50.0	0	0
Sutter Buttes State Park	40	0	0	1	2.5
Sagehen Research Station	221	0	0	1	0.4
Siskiyou County (roadside only);	4	4	100.0	0	0
Sonoma	1	0	0	1	100.0
Samuel P. Taylor State Park	171	1	0.6	21	12.3
Trinity	5	5	100.0	0	0
Sacramento Valley	3	3	100.0	0	0
Willow Creek	10	4	40.0	0	0
Yolo	15	2	13.3	13	86.7

Table 4. Tick species and stage recovered from rodents throughout California*

	Dermacentor sp.			*D. occidentalis*			*Ixodes* sp.			*I. angustus*			*I. ochotonae*			*I. pacificus*			*I. spinipalpis*			*I. woodi*		
Host Species	A	N	L	A	N	L	A	N	L	A	N	L	A	N	L	A	N	L	A	N	L	A	N	L
C. californicus	0	0	0	0	0	0	0	0	0	0	0	0	0	0	0	0	0	1	0	0	0	0	0	0
G. sabrinus	0	1	0	0	0	0	0	0	0	0	0	0	0	0	0	0	0	2	0	0	0	0	0	0
M. californicus	0	0	0	0	13	8	0	0	0	2	4	0	0	0	0	0	0	1	0	0	0	0	0	0
M. musculus	0	0	0	0	0	0	0	0	0	0	3	0	0	0	0	0	0	0	0	0	0	0	0	0
N. fuscipes	0	1	0	0	3	0	1	3	1	3	11	0	0	10	1	1	2	3	9	2	1	3	0	0
P. boylii	0	0	0	0	0	0	0	0	0	0	0	0	0	0	0	0	0	0	0	0	1	0	0	0
P. californicus	0	0	0	0	1	3	2	0	1	5	25	7	0	0	0	0	2	4	0	0	2	3	0	0
P. maniculatus	0	2	1	0	1	7	10	0	1	3	31	5	6	0	0	7	3	20	0	0	4	2	0	0
P. truei	0	0	0	0	0	1	1	0	0	0	0	0	1	0	0	0	0	0	0	0	0	0	0	0
R. rattus	0	0	0	0	0	0	0	0	0	0	5	0	0	0	0	0	0	0	0	0	0	0	0	0
R. megalotis	0	0	0	0	0	0	0	0	0	1	0	0	0	0	0	0	0	0	0	0	0	0	0	0
S. carolinensis	0	0	0	0	0	0	0	0	0	1	0	1	0	0	0	2	0	0	0	0	0	0	0	0
S. griseus	0	0	0	0	0	0	0	0	0	0	1	0	0	0	0	0	6	19	0	3	0	0	0	0
Sp. beecheyi	0	0	0	0	0	0	0	0	0	0	0	0	2	0	0	0	1	0	0	0	0	0	0	0
T. merriami	0	0	0	0	0	0	0	0	0	0	0	0	1	0	0	0	0	0	0	0	0	0	0	0
T. ochrogenys	0	1	1	0	0	0	1	1	1	2	12	3	6	0	0	0	8	85	0	1	0	1	0	0
T. sonomae	0	0	0	0	0	0	0	0	0	0	0	2	0	0	0	0	0	0	0	0	0	0	0	0
Tamiasciurus douglasii	0	0	0	0	0	0	0	0	0	0	0	1	0	0	0	0	0	0	0	0	0	0	0	0
Sorex sp.	0	0	0	0	0	0	0	0	0	0	0	0	0	0	0	0	0	4	0	0	0	0	0	0
Total	0	5	2	0	18	19	15	4	4	17	92	19	16	1	1	10	22	139	9	6	8	9	0	0

*Data not shown: 1 *I .hearlei* adult was observed on a northern flying squirrel; 3 adult *I. jellisoni* on deer mice. A = adult; N = nymph; L = larvae.

Table 5. Ticks collected on rodents from 8 different study areas in California*

	Dermacentor sp.			*D. occidentalis*			*Ixodes* sp.			*I. angustus*			*I. ochotonae*			*I. pacificus*			*I. spinipalpis*			Total		
Study areas	A	N	L	A	N	L	A	N	L	A	N	L	A	N	L	A	N	L	A	N	L	A	N	L
Big Basin State Park	0	0	0	0	0	0	2	0	1	0	4	1	1	0	0	6	0	1	0	0	0	12	4	3
Rural Yolo County	0	0	0	0	0	0	0	3	1	0	0	0	0	10	1	0	0	0	0	0	0	0	13	2
Humboldt Redwoods State Par	0	0	0	0	0	0	4	0	0	1	13	0	9	0	0	1	3	12	0	2	0	17	18	12
Hendy Woods State Park	0	3	1	0	0	0	4	3	0	2	19	3	5	0	0	1	10	97	7	1	3	21	36	104
King Range National Conservation Area	0	0	0	0	0	3	0	0	0	0	1	2	0	0	0	0	0	0	0	0	0	0	1	5
Morro Bay regional communities	0	0	0	0	16	16	0	0	0	13	49	10	0	0	0	0	3	3	1	0	5	14	68	34
Samuel P. Taylor State Park	0	2	1	0	0	0	5	0	1	0	13	2	1	0	0	3	2	8	0	1	0	15	18	12
Sutter Buttes State Park	0	0	0	0	1	0	0	0	0	0	0	0	0	0	0	0	0	1	0	0	0	0	1	1

*Data not shown: 3 I. jellisoni adults were recovered from Samuel P. Taylor State Park; adult I. woodi were collected from Big Basin State Park (3), Humboldt Redwoods State Park (2), Hendy Woods State Park (2), and Samuel P. Taylor State Park (4). A = adult; N = nymph; L = larvae,
